# Determination of the effective dose of bone marrow mononuclear cell therapy for bone healing in vivo

**DOI:** 10.1007/s00068-020-01331-2

**Published:** 2020-02-28

**Authors:** Maren Janko, Sabrina Pöllinger, Alexander Schaible, Marlene Bellen, Katrin Schröder, Myriam Heilani, Charlotte Fremdling, Ingo Marzi, Christoph Nau, Dirk Henrich, René D. Verboket

**Affiliations:** 1 Department of Trauma-, Hand- and Reconstructive Surgery, Hospital of the Goethe-University, Frankfurt am Main, Germany; 2 Center of Physiology, Cardiovascular Physiology, Hospital of the Goethe-University, Frankfurt am Main, Germany

**Keywords:** BMC, Bone defect, Tissue engineering, Critical size

## Abstract

**Introduction:**

Cell-based therapy by bone marrow mononuclear cells (BMC) in a large-sized bone defect has already shown improved vascularization and new bone formation. First clinical trials are already being conducted. BMC were isolated from bone marrow aspirate and given back to patients in combination with a scaffold within some hours. However, the optimal concentration of BMC has not yet been determined for bone healing. With this study, we want to determine the optimal dosage of the BMC in the bone defect to support bone healing.

**Material and methods:**

Scaffolds with increasing BMC concentrations were inserted into a 5 mm femoral defect, cell concentrations of 2 × 10^6^ BMC/mL, 1 × 10^7^ BMC/mL and 2 × 10^7^ BMC/mL were used. Based on the initial cell number used to colonize the scaffolds, the groups are designated 1 × 10^6^, 5 × 10^6^ and 1 × 10^7^ group. Bone healing was assessed biomechanically, radiologically (µCT), and histologically after 8 weeks healing time.

**Results:**

Improved bone healing parameters were noted in the 1 × 10^6^ and 5 × 10^6^ BMC groups. A significantly higher BMD was observed in the 1 × 10^6^ BMC group compared to the other groups. Histologically, a significantly increased bone growth in the defect area was observed in group 5 × 10^6^ BMC. This finding could be supported radiologically.

**Conclusion:**

It was shown that the effective dose of BMC for bone defect healing ranges from 2 × 10^6^ BMC/mL to 1 × 10^7^ BMC/mL. This concentration range seems to be the therapeutic window for BMC-supported therapy of large bone defects. However, further studies are necessary to clarify the exact BMC-dose dependent mechanisms of bone defect healing and to determine the therapeutically effective range more precisely.

## Introduction

Regeneration of large bone defects is a major problem in trauma surgery and orthopedics. At the moment, the gold standard for their treatment consists of autologous bone chips, taken from the iliac crest. However, this procedure has to deal with donor site complications [1]. In recent times, more and more tissue engineering approaches using cells with osteoinductive potential and different types of scaffolds might circumvent those limitations. The combination of endothelial progenitor cells (EPC) and marrow stromal cells (MSC) with a β-tricalcium phosphate (β-TCP) scaffold was effective in experimental bone healing models [2, 3]. However, MSC/EPC must be culture-expanded prior use which might increase the risk for genetic alterations [4] or contamination with pathogens, as well as it takes time and will be prohibitively expensive. Also, the needed growth factors used for EPC differentiation in vitro such as IGF-1 might be able to support transformation of hematopoietic progenitors [5] from which EPC develop [6]. Due to these risks and limitations, we started investigating the use of bone marrow mononuclear cells (BMC) for bone healing [7–9]. BMC can be harvested and reintroduced to the patient within hours which is more compatible with the clinical requirement for rapid fracture repositioning. There is no need for a long expansion time minimizing the negative effects. Under the heterogeneous mixture of BMC diverse cell types were found i.e. (immature) lymphocytes, (immature) monocytes, and progenitor cell populations. Several types of cells with regenerative potential like precursors of MSC, hematopoietic stem cells (HSC) as a putative source of EPC, and (immature) monocytes were also found [8, 10–13] Our own previous work demonstrated that BMCs seeded on uncoated β-TCP scaffolds [8] (1.3 × 10^6^ BMC/mL) and transplanted into an experimental femur defect, exerted highly beneficial effects on the bone healing response [14, 15] qualitatively comparable to those mediated by cultured MSC and EPC [2, 3]. In our own recently completed phase-I clinical study, we demonstrated that BMC seeded onto pre-implanted β-TCP scaffold in a bone defect was well tolerated and complete bone healing was achieved in all 10 patients after 3 months [9].

However, the optimal concentration of BMC has not yet been determined for bone healing. With this study, we want to determine the optimal dosage of the BMC in the bone defect to support bone healing.

## Materials and methods

### Ethics

Human BMC were isolated from bone marrow aspirates from the iliac crest. Bone marrow samples were kindly provided by the German Red Cross Blood Donor Service Baden-Württemberg-Hessen, Frankfurt, Germany. The use of anonymous bone marrow samples for research purposes was approved by the local ethics committee (Ethik-Kommission des Fachbereichs Medizin der Johann Wolfgang Goethe-Universität, Project number 329/10) and informed consent was acquired from all donors. All animal experiments were performed in accordance with the institutional animal care and oversight committee (Project FK/1091, Regierungspräsidium Darmstadt, Germany), in accordance with German law.

### Scaffolds

Animals were divided into four groups. The first group served as control group, in which only β-TCP was used. The critically-sized femoral defects in all other groups were filled with β-TCP and increasing BMC concentrations.

### BMC isolation and seeding

Human BMC were isolated following the isolation protocol published by Assmus et al. [16]. The bone marrow aspirates were diluted with phosphate-buffered saline (PBS, 1: 3) and mononuclear cells were isolated by density gradient centrifugation with Ficoll (1.077 g/cm^3^, Biochrom, Berlin, Germany) at 800 g for 20 min without brake at room temperature. The cells were washed with 25 mL phosphate-buffered saline (800 g) and counted. Viability was assessed by exclusion dye stain using trypan blue.

500 μL β-TCP scaffold (ChronosO^®^; Synthes, Switzerland) with size 0.7–1.4 mm, porosity 60%, and pore size 100–500 mm for each animal were placed in in individual wells (area = 2 cm^2^) of a 24-well plate (Nunc, Wiesbaden, Germany) using sterile forceps. A number of 1 × 10^6^, 5 × 10^6^ or 1 × 10^7^ BMC in a volume of 500 μL PBS (2 × 10^6^ BMC/mL, 1 × 10^7^ BMC/mL or 2 × 10^7^ BMC/mL) was dripped slowly on the scaffolds and incubated for 5 min at 37 °C. Based on the initial cell number used to colonize the scaffolds, the groups are designated 1 × 10^6^, 5 × 10^6^ and 1 × 10^7^ group. After incubation, the cell suspension not absorbed by the scaffold was removed and dripped once again over the material, followed by a second incubation of 5 min. The approximate volume of the bone defect is 100 µL reaching cell numbers of 2 × 10^5^, 1 × 10^6^ and 2 × 10^6^ in the defect.

### Animal care and BMC transplantation

For the critical-sized defect model, male athymic LOU/MRj-Foxn1rnu/rnu rats were used. Animals were 8 weeks old and weighing approximately 250–300 g. The animals were purchased from Janvier (Janvier, France) and four animals per cage were housed with food and water ad libitum, in temperature (15–21 °C) with air flow- and light-controlled (14 h day, 10 h night). Animal wellbeing was ascertained daily during the first week after surgery and weekly thereafter.

A general anesthesia with 2 mL of a mixture of Ketavet (70 mg/kg) and Rompun (10 mg/kg) was given intraperitoneally. Under aseptic conditions, the right rat femur was dissected. A five-hole plate *(CompactHand, Synthes, Dubendorf, Switzerland)* was then positioned on the femur and secured in place with four 1.3 mm cortical screws *(CompactHand*,* Synthes)*, leaving the middle hole free. Using a Gigli saw a 5 mm defect of the femur was created under the free middle hole of the plate. A 5 mm defect in a rat femur was shown to be a critical size defect in previous studies [17, 18] The scaffolds were implanted into the segmental defects and the wound was closed in two layers with continuous subcutaneous stiches using a 4/0-monofilament nylon suture.

As postoperative analgesia, the animals received 2.6 mg/kg Carprofen s.c. on operation and first following day and overlapping 2.5 mg/100 mL Tramadol in the drinking water as pain treatment over the following 5 days. The rats were housed under standard conditions and nutrition for 8 weeks.

The animals were sacrificed after 8 weeks by inhalation anesthesia and following intra-cardial pentobarbital injection (150 mg/kg). Femora were explanted and the ambient tissue was removed. The explanted bones were examined regarding signs of infection or tumors and the firm fit of the implanted screws was checked.

### Biomechanical characterisation

Mechanical testing was performed by a standardized three-point bending test using a material testing device (Zwicki-line 5.0; Zwick-Roell, Ulm, Germany). The bone was placed onto the device to measure the stability of the bone in an anterior/posterior direction. The degree of displacement in mm at the highest pressure reached was noted. Stiffness [slope of the elastic deformation part of the load/deformation curve] was then calculated using the Testexpert-II software (Zwick-Roell).

### µCT analysis

Bone mineral density was assessed via µCT analysis with a high-resolution in-vivo-micro-CT Skyscan 1176 (Bruker AXS, Karlsruhe, Germany). The long axis of the femur was lined up orthogonally to the axis of the X-ray beam (Al 0.5 mm; voltage: 50 kV; current: 500μA; frame average: 7; rotation ra.: 180; rotation st.: 0.5). The region of interest was placed on the defect and the isotropic voxel size was 18 µm^3^. Two-dimensional CT-images were scanned of each bone then reconstructed using a standard back convolution procedure and saved in 3D arrays.

### Histological assessment of callus formation

Histomorphometric analysis was performed to assess callus formation and bone maturation. Movat pentachrome, CD68, α-SMA and osteocalcin immunostaining of decalcified bone sections were performed as described in [14, 15, 19, 20]. In brief, for histological evaluation of bone maturation, bones were carefully defrosted and fixed in Zinc-Formal-Fixx, 10% over 20 h (Thermo Electron, Pittsburgh, USA) followed by decalcification for 14d in 0.25 M Trizma base (Sigma-Aldrich, Taufkirchen, Germany) and 10% EDTA (Sigma-Aldrich), pH-value 7.4. After decalcification bones were paraffin embedded and longitudinal sections (3 µm) were taken. Movat pentachrome staining of paraffin embedded histological slides was performed as published by Garvey et al. [21] using a staining kit according to the manufacturer’s instructions (Morphisto, Frankfurt, Germany).

Callus maturation was measured using immunohistochemistry via detection of osteocalcin, vascularization via staining of α-smooth muscle actin (α-SMA) and presence of macrophages via staining of CD68. The sections were incubated with monoclonal mouse anti-rat osteocalcin (1 h, 10 µg/mL, 75 µL/slide, clone 1A4, Abcam, Cambridge, UK), monoclonal mouse anti-rat α-SMA (1 h, 2 µg/mL, 75 µL/slide, clone OC4-30, Abcam,) or monoclonal anti Rat CD68 (clone KP1, 1 h, final concentration 2 µg/mL, 1 h, Abcam). As secondary antibody, a polyclonal HRP conjugated anti-mouse IgG (Simple Stain Rat MAX PO, Nichirei, Tokyo, Japan) was applied for 30 min followed by incubation with 3-amino-9-ethylcarbazole (AEC, Sigma) following the instructions of the manufacturer. Finally, a counterstain with hematoxylin was performed. An independent observer blinded to the group setup analyzed the samples. All slides were analyzed using light microscopy (Axioobserver Z1, Zeiss, Gottingen, Germany; Biorevo BZ-9000, Keyence, Neu-Isenburg, Germany) in combination with a computer-supported imaging picture analysis system (Axiovision, Zeiss; BZII-Analyser, Keyence). High resolution images depicting the whole defect zone in each case were created by automated stitching of multiple single frames covering the whole defect using the software BZII Analyzer (Keyence). New bone formation, cartilaginous tissue area, osteocalcin positive area, and a-SMA positive blood vessels were then analyzed in the defect site using the software ImageJ (https://imagej.nih.gov/ij/) and the relative tissue positive area of the entire defect zone was calculated. Since intensity of osteocalcin staining in the various tissues/biomaterials does not allow for fully automated image analysis, area of osteocalcin positive new bone tissue was marked by an independent observer using the polygon tool of ImageJ and the relative osteocalcin positive area of the entire defect zone was calculated. The mean number of CD68-positive macrophages and CD68 giant cells were assessed by counting CD68-positive cells/giant cells in three non-overlapping microscopic fields of view covering a proximal, central and distal area of the bone defect at 200-fold magnification. Mean values per animal were calculated which served as data for subsequent statistical analysis. In all cases the observer was blinded to the group allocation.

### Statistics

Results are presented as box plots of the median in diagrams or as mean and standard deviation in the description of the results. A non-parametric Kruskal–Wallis-test with Bonferoni–Holm corrected Conover–Iman post hoc analysis was used for comparisons between the groups using the statistical software Bias 11.10 (Epsilon-Verlag, Darmstadt, Germany). *P *values < 0.05 indicate statistical significance.

## Results

### Animal care

All animals were included in the evaluation. Screw loosening did not occur and no macroscopically visible side effects were recorded. At the time of sacrifice, the weight of the animals was comparable in all groups. The animals in the β-TCP group weighed 623.5 g ± 33.2 g, in the β-TCP + 1 × 10^6^ BMC group 621.9 g ± 29.5 g, in the β-TCP + 5 × 10^6^ BMC group 627.4 g ± 35.2 g and in the β-TCP + 1 × 10^7^ BMC group 618.7 g ± 22.6 g.

### Low bending stiffness in all treatment groups

**Fig. 1:**
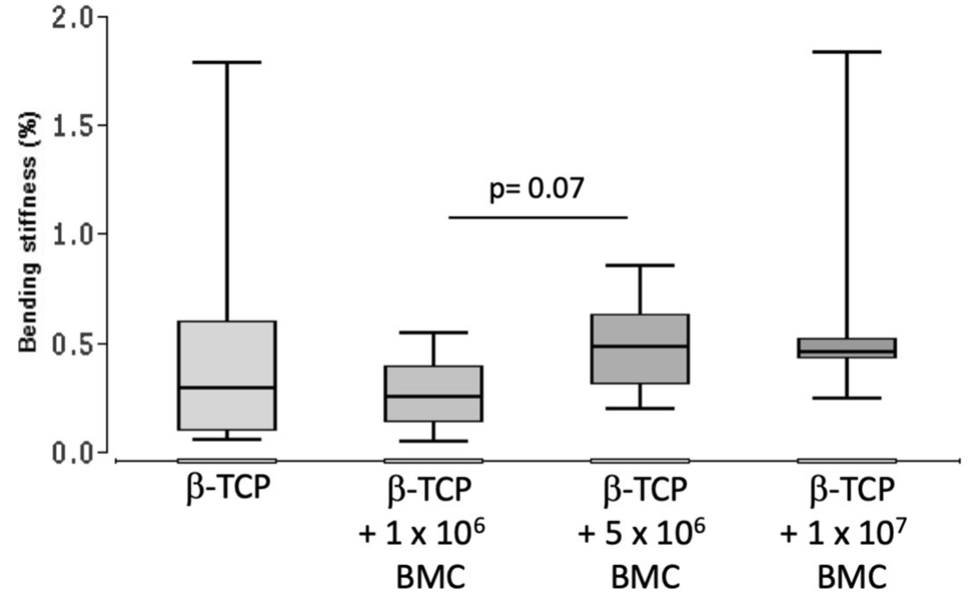
Bending stiffness of the defect zone in β-TCP, β-TCP + 1 × 10^6^ BMC, β-TCP + 5 × 10^6^ BMC and β-TCP + 1 × 10^7^ BMC groups. Biomechanical properties of the defect zone were measured by means of three-point bending test 8 weeks after transplantation. A trend to a higher bending stiffness in β-TCP + 5 × 10^6^ BMC vs. β-TCP + 1 × 10^6^ BMC was noted (*P* = 0.07)

Bending stiffness in % of the contralateral healthy femur was evaluated (Fig. 1). Similar results were observed in the β-TCP and β-TCP + 1 × 10^6^ BMC group (0.32% ± 0.6% and 0.25% ± 0.16%). A trend towards higher bending stiffness was observed in the β-TCP + 5 × 10^6^ BMC group vs. the β-TCP + 1 × 10^6^ BMC group (0.48% ± 0.12% and 0.25% ± 0.16%; *P* = 0.07). No significances between the groups were detected.

### Histologically evaluated new bone formation is increased in animals of the 5 × 10^6^ BMC group

**Fig. 2:**
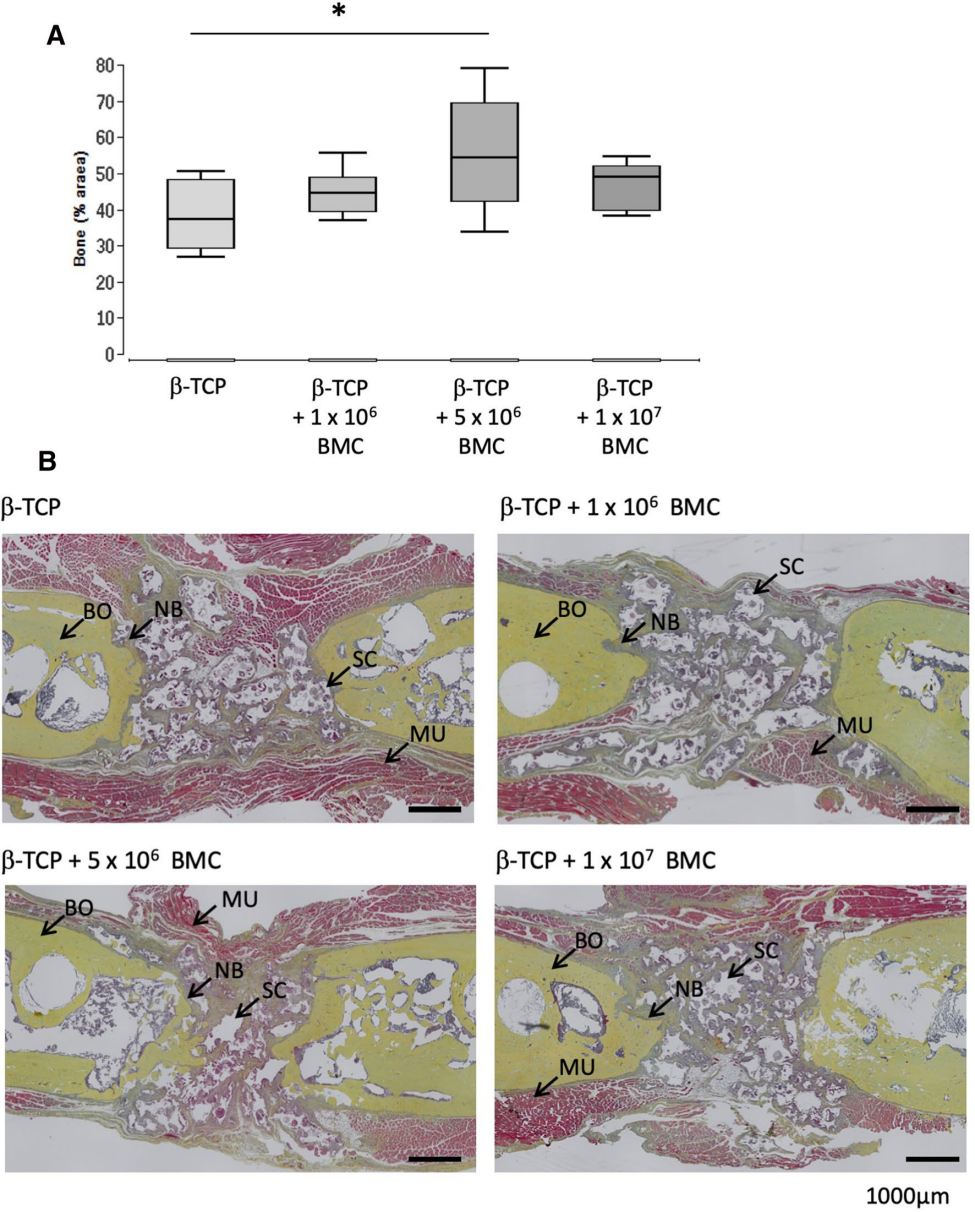

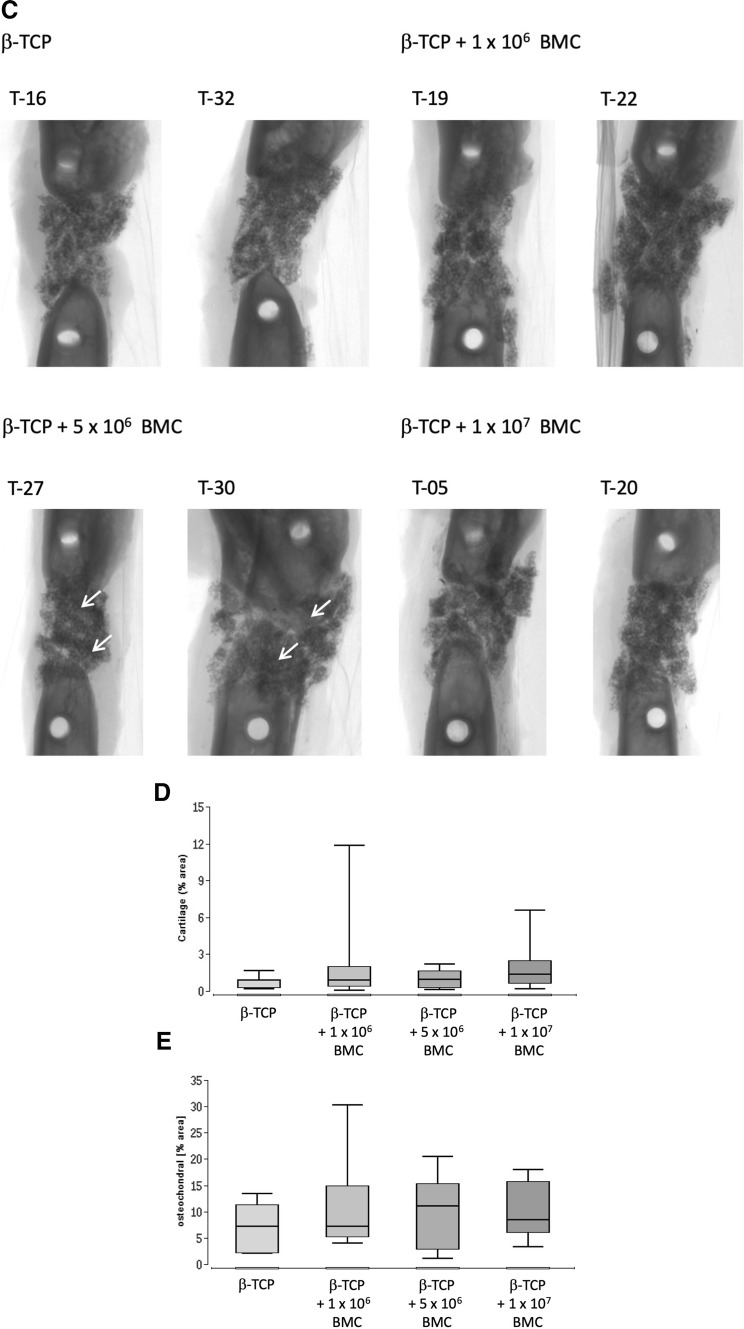
Percentage of bone tissue (**a**) in the defect area as assessed by histomorphometric analysis of Movat’s pentachrome stained histological slices 8 weeks after surgery. Representative images of Movat’s pentachrome staining (**b**). The images provide an overview of the whole defect. Bone tissue appears yellow, cartilaginous tissue appears cyan. BO = bone, NB = newly formed bone tissue, MU = muscle, SC = β-TCP scaffold. Black bars represent 1000 µm, representative images of µCT analysis (**c**) area with new bone formation marked with white arrow. Percentage of Cartilage (**d**) and osteochondral differentiation (**e**) of Movat’s pentachrome stained histological slices 8 weeks after surgery. *P < 0.05

The percentage of bone tissue, cartilage and osteochondral differentiation in the defect area was assessed by histomorphometric analysis of Movat’s pentachrome stained histological slices at 8 weeks after surgery. Newly formed bone had replaced 37.3% ± 8.7% of the bone defect in the β-TCP group, compared to 44.6% ± 5.6% in the β-TCP + 1 × 10^6^ BMC group. In the β-TCP + 5 × 10^6^ BMC group, newly formed bone covered 54.3% ± 14.6% of the defect area (*P* < 0.05 vs. β-TCP group). Newly formed bone substance covered 48.9% ± 5.8% of the defect area in the β-TCP+ 1×10^7^ BMC group (Fig. 2).

In none of the groups, bone was radiologically healed. In the β-TCP + 5 × 10^6^ BMC group, we were able to see callus formation in the µCT scans, but no complete ossification. The callus formation was not observable in the other groups (Fig. 2). In all of the µCT scans bone formation was at a very early stage.

The mean cartilage formation was 0.9% ± 0.4% of the defect area in the β-TCP group, 0.88% ± 3.5% in the β-TCP + 1 × 10^6^ BMC group, 0.95% ± 0.67% in the β-TCP + 5 × 10^6^ group and 1.35% ± 1.8% in the β-TCP + 1 × 10^7^ BMC group. As in the assessment of osteochondral differentiation, no significant differences between the groups were detectable (Fig. 2).

### Bone maturation: increased BMD in animals receiving 1 × 10^6^ BMC, no changes in osteocalcin expression

**Fig. 3:**
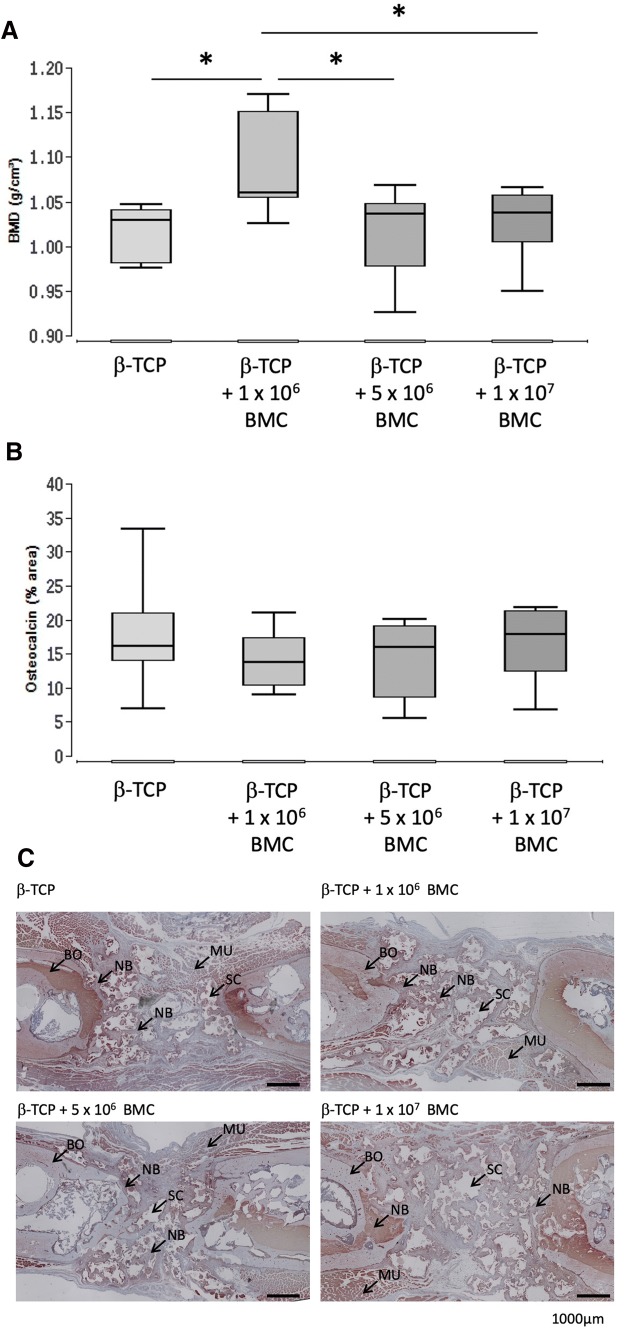
Bone maturation in the different groups. Bone mineral density (BMD) assessed by µCT (**a**) and osteocalcin positive areas assessed by histomorphometric analysis (**b**) of stained histological slices 8 weeks after surgery. Representative images of osteocalcin staining in the different groups are shown (**c**). Images provide an overview of the whole defect, osteocalcin-positive area demonstrates a brown color. BO = bone, NB = newly formed bone tissue, MU = muscle, SC = β-TCP scaffold. Black bars represent 1000 µm, *P < 0.05

Bone mineral density (BMD) in g/cm^3^ was assessed by µCT (Fig. 3). The significantly highest BMD was found in the β-TCP + 1 × 10^6^ BMC group 1.06 ± 0.07 g/cm^3^ (*P* < 0.05 vs. β-TCP group, vs. β-TCP + 5 × 10^6^ BMC group and vs. β-TCP + 1 × 10^7^ BMC group). No significant differences were observed between the other groups.

The percentage of bone tissue and cartilage in the defect area was assessed by histomorphometric analysis of osteocalcin stained histological slices at 8 weeks after surgery. Osteocalcin positive areas were visible in 16.2% ± 7.4% of the bone defect in the β-TCP group, compared to 13.8% ± 3.47% in the β-TCP + 1 × 10^6^ BMC group, 15.9% ± 5.32% in the β-TCP + 5 × 10^6^ BMC group and 17.8% ± 4.9% in the β-TCP + 1 × 10^7^ BMC group. No significant differences were detectable (Fig. 3).

### Vascularization is not altered in dependency of the BMC-dose

**Fig. 4:**
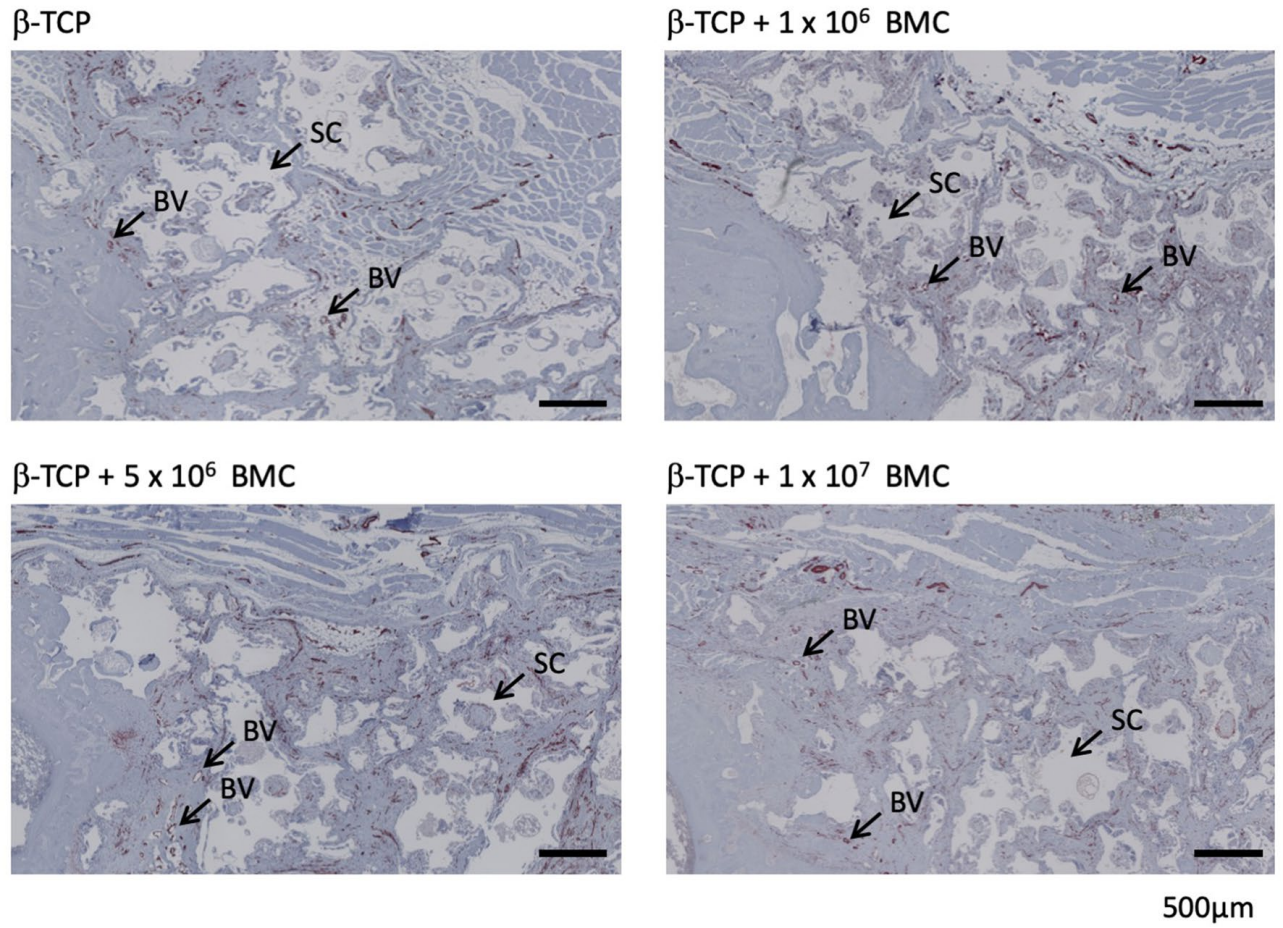
Vascularization in control and treatment groups. Representative images of α-SMA staining. The sectional enlargements of the bone defect were uniformly aligned with the proximal cortex. α-SMA-positive structures appear brownish and are marked with BV. BV = blood vessel, SC = β-TCP scaffold. Black bars represent 500 µm

The percentage of vascularization in the defect area was assessed by histomorphometric analysis of α-SMA stained histological slices at 8 weeks after surgery. α-SMA positive areas were detectable in 2.56% ± 0.89% of the bone defect in the β-TCP group, compared to 2.08% ± 1.09% in the β-TCP + 1 × 10^6^ BMC group. In the β-TCP + 5 × 10^6^ BMC group α-SMA positive areas covered 2.35% ± 0.67% of the defect area, in the β-TCP + 1 × 10^7^ BMC group 2.58% ± 1.4%; no significant differences were detectable (Fig. 4).

### Numbers of macrophages and giant cells in the bone defect are not BMC-dose dependent

Presence of macrophages as CD68^+^ cells and giant cells in the defect zone was assessed by cell counting in histological slices 8 weeks after surgery. Values are presented as number of cells per high power field. No significant differences in macrophage numbers or giant cell accumulation were detectable between the groups.

## Discussion

The therapeutic effect of the concentration of BMC on the healing of a large (5 mm) rat femoral bone defect filled with β-TCP supplemented with increasing BMC doses was investigated. The different concentrations of 1 × 10^6^, 5 × 10^6^ and 1 × 10^7^ BMC per 500 µL volume were compared to a filling of the bone defect solely with β-TCP. Improved bone healing parameters were noted in the 1 × 10^6^ and 5 × 10^6^ BMC groups. A significantly higher BMD was observed in the 1 × 10^6^ BMC group compared to the other groups. Histologically a significantly increased bone growth in the defect area was observed in group 5 × 10^6^ BMC. This finding could be supported radiologically.

### BMC in bone regeneration

Hisatome et al. reported the use of BMC for bone healing in 2005 for the first time [22]. The group examined “whether the transplantation of autologous BMC can augment neovascularization and bone regeneration in femoral bone defects of rabbits” [22]. Sun et al. also investigated the use of BMC, focusing on the vascularization of bone defects [23]. As with Hisatome et al. the application of BMC into the defect zone led to improved bone healing and higher neovascularization. The results substantiated the idea of BMC-supported bone healing also in humans [22, 23]. Cells within a BMC preparation holding an evidentially regenerative potential are (immature) monocytes and hematopoietic stem cells (HSCs) as putative source of EPCs and precursors of MSCs [6, 12, 24, 25]. The first clinical trials on the use of BMC in proximal plate-stabilized humeral fractures have already been conducted and could demonstrate the safe application of the cells in humans [9], further phase 2 results are still pending.

Uncoated β-TCP was used as scaffold in the present study. This decision was based on previous results [8] which showed a similar seeding efficiency of BMC between coated and uncoated scaffolds. Based on these results, uncoated scaffolds are also used for the current clinical trial (EudraCT No. 2012-004,037-17) [7, 9]. Great efforts have been made to identify suitable carrier materials [7, 26–32], but to our knowledge, systematic analyses to find the optimal cell dose have not yet been performed for cell-assisted therapy of large bone defects.

### Dose effect study

This work was the first attempt to conduct a dose finding study for BMC-assisted bone healing. Even after intensive literature research it was not possible to find dose information for cell-assisted bone healing therapies, whereas the timing of application [33] and the preparation of the cells, especially MSC [4], are well investigated.

1 × 10^6^ BMC per 500–1000 µL scaffold volume are used in most studies analyzing the effect of BMC in bone defects under various conditions [4, 9, 14, 33, 34]. This BMC concentration range, being effective in bone healing, was originally derived from the use of BMC in cardiology, where a number of 7.3 × 10^6^/10 mL BMC in intracoronary application was considered safe in clinical studies [25]. When considering the seeding of cells on scaffolds, which is necessary for use in bone healing, it should be noted that only about 90% of the cells reach the defect site due to cell losses during seeding [8].

In this work dose-dependent effects of BMC related to bone healing were demonstrated. Conversely to prior work, it was shown that transplantation of cell free β-TCP leads to completely biomechanically unstable bone, whereas β-TCP augmented with BMC improved biomechanical characteristics (Fig. 1) [3, 14]. Other bone healing parameters were also improved in a dose dependent manner. The highest BMD was achieved in the 1 × 10^6^ BMC group (Fig. 3). In combination of those results with histologically and radiologically evaluated bone mass increase in the 5 × 10^6^ BMC group, the optimal concentration of BMC is probably in the range of 1–5 × 10^6^ BMC/500 µL scaffold volume. According to the results shown, it can be assumed that the number and effect of the cells used corresponds to a bell-shaped optimum curve. Higher concentrations lead to a decrease in new bone formation and of BMD (Figs. 2, 3).

**Fig. 5:**
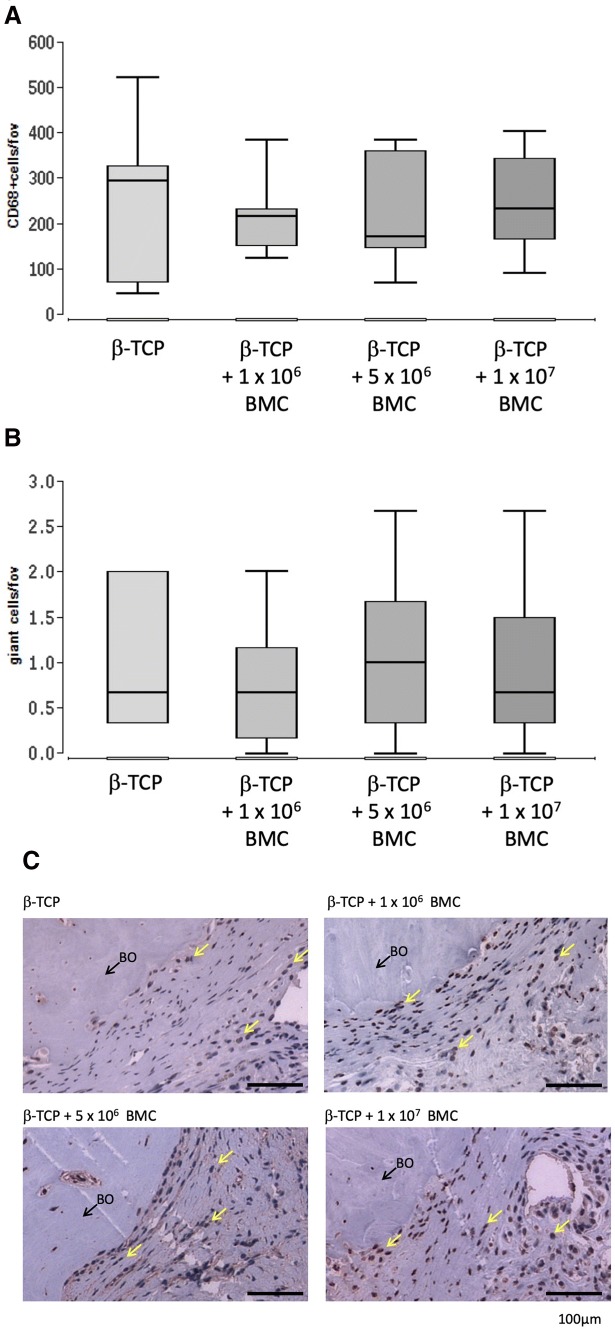
Macrophages in the defect zone assessed by histomorphometric analysis of CD68 stained histological slices 8 weeks after surgery. Mean number of CD68^+^ cells per field of view (fov) at 200-fold magnification (**a**) and mean number of giant cells per fov (**b**) are presented. Representative images of CD68 staining (**c**). The images provide a detailed enlargement of the whole defect. Yellow arrows indicate monocytes. BO = bone. Black bars represent 100 µm

Foreign body reactions against the implanted biomaterial are performed by giant cells consisting of fused macrophages [35]. To check whether the additional entry of monocytes through BMC leads to an increased foreign body reaction that would inhibit bone healing, the concentration of macrophages and giant cells was analyzed histologically as a function of the BMC dose (Fig. 5). However, neither differences in macrophage concentration (CD68-positive), nor in the concentration of giant cells, which in addition could only be observed in small numbers, could be found between the different treatment groups. These findings suggest that even a high concentration of BMC does not demonstrably increase the foreign body response to β-TCP, and that therefore other mechanisms are more effective in reducing bone healing at high BMC concentrations. These suspected inflammatory processes might have been relevant in the early phase of bone defect healing and could therefore not be detected in this study.

### Limitations of the study

All analyses were carried out after an 8-week healing period, i.e. early processes with influence on bone healing cannot be observed.

In principle, this study gives a rough idea of the effective BMC dose needed to induce and enhance induced bone healing. A finer graded concentration range may lead to a better estimation of the effective BMC concentration.

## Conclusion

In this study, the first dose effect study for BMC in bone healing was performed. It was shown in a 5 mm femur defect model of the athymic nude rat that the effective dose of human BMC for bone defect healing ranges from 2 × 10^6^ cells to 1 × 10^7^ cells per mL volume. This concentration range seems to be the therapeutic window for BMC-supported therapy of large bone defects filled with a scaffold in a stable healing situation. However, further studies are necessary to clarify the exact BMC-dose dependent mechanisms of bone defect.
